# Ultrasound-guided fine needle aspiration thyroglobulin in the diagnosis of lymph node metastasis of differentiated papillary thyroid carcinoma and its influencing factors

**DOI:** 10.3389/fendo.2024.1304832

**Published:** 2024-03-11

**Authors:** Xuejiao Su, Lei Shang, Can Yue, Buyun Ma

**Affiliations:** ^1^ Department of Medical Ultrasound, West China Hospital of Sichuan University, Chengdu, Sichuan, China; ^2^ Department of Medical Ultrasound, XinQiao Hospital, Army Medical University, Chongqing, China

**Keywords:** fine needle aspiration, fine needle aspiration thyroglobulin, differentiated thyroid cancer, lymph node metastasis, serum thyroglobulin, serum thyroglobulin antibodies, large or high volume lymph node metastasis

## Abstract

**Background:**

Ultrasound-guided fine needle aspiration thyroglobulin (FNA-Tg) is recommended for the diagnosis of lymph node metastasis (LNM) in differentiated thyroid cancer (DTC), but its optimal cutoff value remains controversial, and the effect of potential influencing factors on FNA-Tg levels is unclear.

**Method:**

In this study, a retrospective analysis was conducted on 281 patients diagnosed with DTC, encompassing 333 lymph nodes. We analyze the optimal cutoff value and diagnostic efficacy of FNA-Tg, while also evaluating the potential influence of various factors on FNA-Tg.

**Results:**

For FNA-Tg, the optimal cutoff value was 16.1 ng/mL (area under the curve (AUC)= 0.942). The optimal cutoff value for FNA-Tg/sTg was 1.42 (AUC = 0.933). The AUC for FNA combined with FNA-Tg yielded the highest value compared to other combined diagnostic methods (AUC = 0.955). It has been found that serum thyroglobulin (sTg) is positively correlated with FNA-Tg (Rs = 0.318), while serum thyroglobulin antibodies (sTgAb) is negatively correlated with FNA-Tg (Rs = -0.147). In cases where the TNM stage indicated N1b, the presence of large or high volume lymph node metastasis(HVLNM), lymph node lateralization/suspicion (L/S) ratio ≤ 2, ultrasound findings indicating lymph node liquefaction, calcification, and increased blood flow, patients with coexisting Hashimoto’s thyroiditis (HT), a tumor size ≥10 mm, and postoperative pathology confirming invasion of the thyroid capsule, higher levels of FNA-Tg were observed. However, the subgroup classification of DTC and the presence or absence of thyroid tissue did not demonstrate any significant impact on the levels of FNA-Tg.

**Conclusion:**

The findings of this study indicate that the utilization of FNA in conjunction with FNA-Tg is a crucial approach for detecting LNM in DTC. TNM stage indicated N1b, the presence of HVLNM, the presence of HT, lymph node L/S ratio, liquefaction, calcification, tumor diameter, sTg and sTgAb are factors that can impact FNA-Tg levels.

In the context of clinical application, it is imperative to individualize the use of FNA-Tg.

## 1 Introduction

DTC, comprising papillary thyroid cancer(PTC) and follicular thyroid cancer, represents the predominant histological subtype of thyroid malignancies, constituting approximately 85-90% of cases (1, 2). Despite the generally slow progression of DTC in terms of clinical and biological behavior, the occurrence of LNM is not uncommon. This metastasis has been found to be closely linked to both recurrence and prognosis (3, 4), thereby significantly influencing the surgical approach chosen for patients. Consequently, the accurate identification of LNM and the exclusion of benign reactive lymphadenopathy are crucial for enhancing patient survival rates and preventing unnecessary lymphadenectomy procedures.

Currently, ultrasound and FNA are frequently employed diagnostic techniques for evaluating LNM in clinical settings. Nevertheless, the intricate neck anatomy contributes to significant variability (ranging from 33% to 70%) in the sensitivity of conventional ultrasound, thereby limiting its diagnostic effectiveness (5). Furthermore, the diagnostic capacity of FNA diminishes in cases where lymph nodes are small, contain lymphocytic infiltrates or necrosis, and lack epithelial components in cyst aspirates (6) exhibiting insufficient cell numbers, or suffering from inadequate sample representation (7). Recent research has demonstrated that the measurement of Tg from FNA eluates is a reliable method for detecting cervical LNM, exhibiting a commendable diagnostic efficacy (3, 8), particularly for smaller lymph nodes (9). The American Thyroid Association (ATA) management guidelines advocate for the incorporation of FNA-Tg measurement as a supplementary tool to FNA cytology when encountering suspicious LNs during ultrasound examination (10). However, the determination of the optimal cutoff value for FNA-Tg remains a subject of controversy, with various methods suggesting values ranging from 0.2 to 36 ng/mL (11). Standardization of the FNA-Tg procedure has yet to be achieved.

Several factors, including serum thyroid-stimulating hormone (TSH), sTgAb, sTg, and the presence or absence of thyroid tissue in situ, may contribute to the variability of FNA-Tg results (3, 9). While some researchers have reported that the clinical manifestations of FNA-Tg are unaffected by sTgAb and TSH levels (12, 13), it has also been suggested that the presence of thyroid tissue, sTg, and sTgAb can significantly impact the diagnostic value of FNA-Tg (14–17).

HVLNM is defined as the presence of more than five metastatic lymph nodes (10). Previous studies have demonstrated that patients with HVLNM experience worse outcomes compared to those with small-volume LNM, including higher rates of recurrence and lower disease-free survival rates (18, 19). Consequently, ATA incorporated HVLNM as an intermediate risk factor for recurrence in its 2015 update on recurrence risk stratification. This increased risk is associated with a higher probability of structural recurrence, estimated to be approximately 15% (10). Nevertheless, few studies have investigated the relationship between HVLNM and FNA-Tg levels in suspicious lymph nodes.

Therefore, the objective of this study is to determine the optimal cutoff value and diagnostic efficacy of FNA-Tg, and to compare the diagnostic performance of FNA, FNA-Tg, and their combination for detecting metastasis of DTC in lymph nodes, and to evaluate the potential factors that may influence FNA-Tg levels.

## 2 Method

### 2.1 Patients

In this study, we conducted a retrospective analysis of the pertinents who sought medical care at West China Hospital, Sichuan University, for suspected thyroid cancer, suspected recurrence, or suspected LNM subsequent to thyroid cancer surgery, during the period from January 2018 to May 2021. The inclusion criteria for this analysis were as follows: 1) confirmation of DTC through final pathological diagnosis; 2) preoperative assessment involving ultrasound examination of the thyroid and cervical lymph nodes, FNA of suspicious lymph nodes, FNA-Tg testing, and sTSH, sTg, and sTgAb detection prior to puncture; 3) postoperative procedures including either total or partial thyroidectomy and bilateral cervical lymph node dissection or partial lymph node dissection; 4) postoperative ultrasound follow-up time was exceeding two years. Patients were excluded if they had a final pathologic diagnosis of non-DTC, previous thyroid ablation, or imperfect data.

### 2.2 Ultrasonography

Ultrasonography was conducted using a Mindray (Resona 7), 7-13 MHz high-frequency linear array probe to examine the thyroid and cervical lymph nodes. Two experienced sonographers with more than 10 years of expertise in thyroid subgroups independently evaluated the sonographic characteristics of suspicious nodules and lymph nodes. The following information was gathered: the size of the suspicious mass, the ultrasound image characteristics of the suspicious lymph nodes (including L/S ratio, calcification, liquefaction, focal hyperechoic regions are observed within the cortex of lymph nodes, blood flow, and other conditions). Blood flow in lymph nodes was assessed using the Adler grading (20).

### 2.3 Ultrasound-guided FNA and FNA-Tg

FNA cytology were primarily conducted using 21-25 gauge needles, although the needle size was adjusted based on the characteristics of the nodules. The needle was inserted parallel to the ultrasound probe. The thyroid samples were obtained through aspiration by retracting the needle for a duration of 5 to 10 seconds. Subsequently, the aspirate was promptly spread onto a slide and preserved in 95% alcohol. The operator evaluated the sufficiency of the specimen in the smear.

After FNA, the needle and syringe were rinsed with 1 ml of saline solution, and the resulting solution was gathered and dispatched to the laboratory for the determination of FNA-Tg (21). FNA-Tg levels were quantified using an immunoradiometric Tg assay(CIS Bio International, located in Saclay, France).

### 2.4 Data collection

The clinical data gathered encompassed patients’ gender, age, and serological-related discoveries. Serum levels of TSH, Tg, and TgAb were assessed utilizing a chemiluminescent immunoassay analyzer prior to puncture. The established reference ranges for sTSH were 0.27-4.2 mU/L, and sTg were 3.5-77 ug/L, and sTgAb < 115 IU/mL. sTgAb (+) represents patient sTgAb ≥115 IU/mL. Staging adhered to the guidelines outlined by the Joint Committee on TNM Staging of Thyroid Cancer in the United States (8th edition, 2017).

### 2.5 Final diagnosis

To ascertain the presence of metastasis in DTC, a definitive malignant diagnosis necessitates confirmation through postoperative pathology. Conversely, a benign diagnosis entails either confirmation through postoperative pathology or a benign repeat FNA result, coupled with unchanged findings from lymph node ultrasound follow-up over a duration of two year (22). Diagnosis of HT is based on postoperative pathology or thyroid function tests combined with ultrasound (positive anti-thyroid peroxidase and/or sTgAb, diffuse enlargement of the thyroid gland and heterogeneous ultrasound echogenicity), with consideration given only to patients exhibiting the presence of thyroid tissue. Furthermore, the assessment of extrathyroidal extension relies on postoperative pathological examination, encompassing the evaluation of infiltration into the thyroid peritoneum, adjacent soft tissues, nerves, blood vessels, and other relevant structures.

### 2.6 Statistical analysis

Statistical analyses were performed using SPSS software version 22.0 (IBM Corp., Armonk, NY, USA). Non-normally distributed continuous variables (age, FNA-Tg, sTSH, sTg, sTgAb, mass size, lymph node size) were presented as medians and interquartile ranges or means and standard deviations; categorical variables were reported as numbers and percentages. The groups were subjected to comparison using the Chi-square test or Mann – Whitney U test. Receiver operating characteristic (ROC) curve analysis was conducted to ascertain the optimal cutoff value for the FNA-Tg, FNA-Tg/sTg by calculating the AUC. Sensitivity, specificity, NPV, PPV, and accuracy were computed to evaluate the diagnostic performance of FNA-Tg, FNA-Tg/sTg, and the combined FNA-Tg and FNA. All statistical tests were two-sided, and a significance level of P < 0.05 was employed.

## 3 Result

### 3.1 Patient characteristics

This study included a cohort of 281 patients (333 lymph nodes), consisting of 176 females and 105 males, with ages ranging from 12 to 80 (40.09 ± 12.72) years. The average maximum tumor diameter was 13.87 ± 9.91 mm (range 3-85 mm), while the maximum lymph node diameter was 12.01 ± 7.05 mm (range 3-63 mm). The pathological classification of DTC comprised 253 cases of classical papillary thyroid carcinoma (PTC), 13 cases of classical PTC combined with follicular PTC, 12 cases of follicular PTC, and 3 cases of follicular carcinoma.

**Figure 1:**
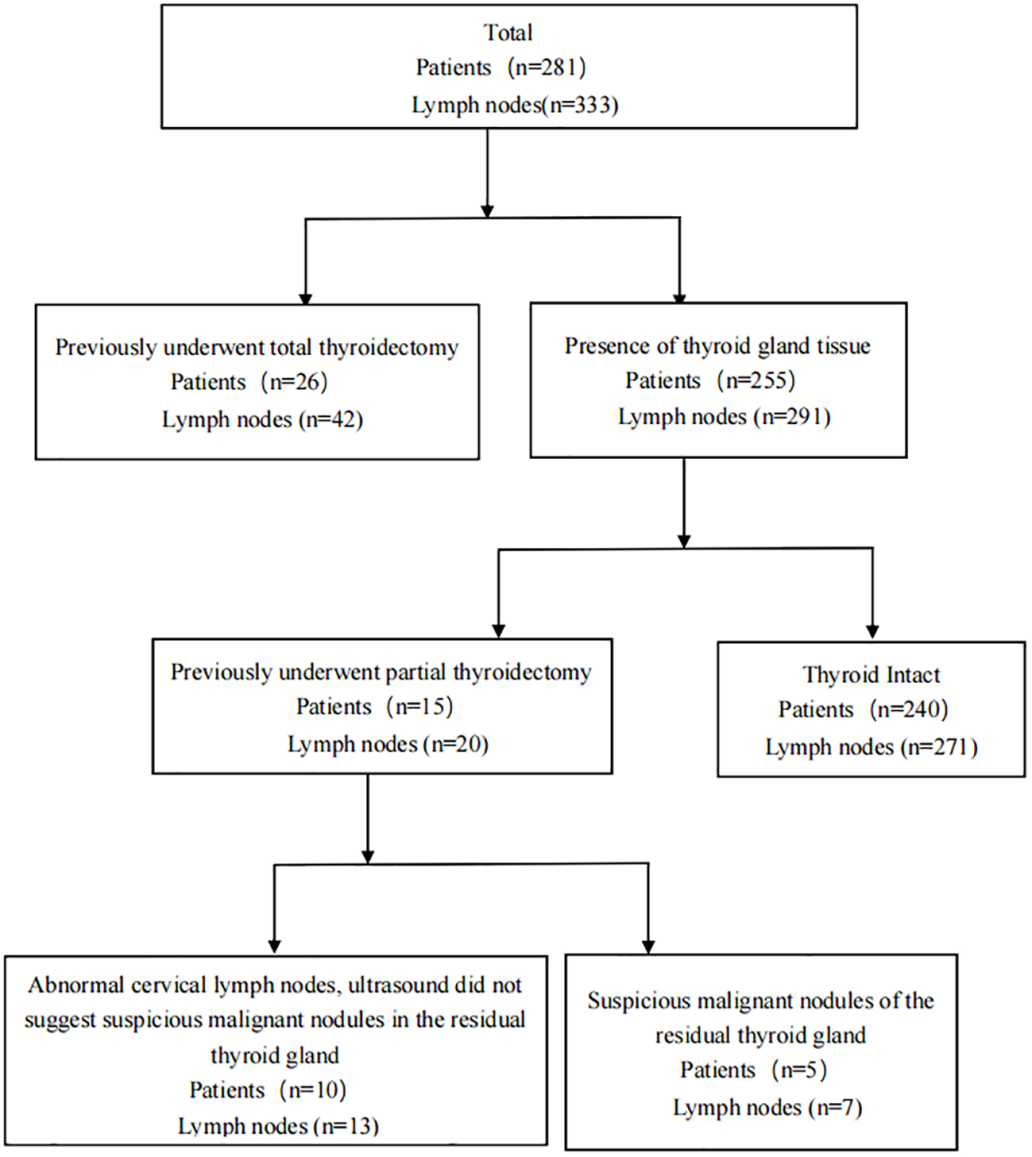
Population detail chart.

A total of 240 patients, who had not undergone any thyroid related surgery in the past, underwent total or hemisection of the thyroid gland, along with ipsilateral or bilateral central lymphadenectomy, as well as other required neck lymphadenectomy procedures. Additionally, 15 patients had previously undergone partial thyroidectomy, with 5 patients(7 lymph nodes)exhibiting suspicious nodules in the remaining thyroid and 10 patients(13 lymph nodes) presenting suspicious metastases in the cervical lymph nodes (ultrasound findings did not indicate the presence of suspected malignant nodules in the remaining thyroid).Furthermore, 26 patients(42 lymph nodes) had previously undergone the necessary lymphadenectomy due to lymph node recurrence subsequent to total thyroidectomy for thyroid cancer (**Figure 1**).Among patients with thyroid suspicious lesions, there were 143 cases of single lesions, 102 cases of multiple lesions. Among the cohort of patients exhibiting thyroid tissue, a total of 188 individuals were found to be devoid of HT, while 67 patients were diagnosed with HT. Additionally, 74 patients (with a total of 88 lymph nodes) underwent both FNA-Tg and FNA-TgAb procedures. Detailed patient information can be found in **Table 1**.

**Table 1:** Characteristic of study population.

Variables	Patients(n=281)	
Sex		
Male	105
Female	176
Age		
<55	232
≥55	49
Prior treatment		
None	240
Unilateral thyroid lobectomy	15
Total thyroidectomy	26
TSH ^a^	2.165(1.965)mU/L
sTg ^a^	13.6(27.183)ng/ml
sTgAb ^a^	16.05(72.9)IU/ml
Hashimoto’s thyroiditis	Patients(n=255)^b^	
No	188	
Yes	67	
Suspected malignant thyroid nodules	Patients(n=245)^c^	
Single	143	
Multiple	102	
Maximum diameter		
<10 mm	100	
10-20 mm	100	
≥20 mm	45	

a Expressed as Mean (interquartile range).

b Among the 281 patients, 26 had a history of total thyroidectomy.

c Out of the total cohort of 15 patients who had previously undergone partial thyroidectomy, 5 sought medical attention due to suspicious thyroid nodules, while 10 presented with suspicious lymph node metastases (as indicated by thyroid ultrasound, which did not detect any suspicious nodules in the remaining thyroid tissue).

### 3.2 Lymph node characteristics

The final diagnosis revealed that 180 lymph nodes were classified as metastatic, while 153 were classified as non-metastatic. All malignant lymph nodes were confirmed through postoperative histopathology analysis. 102 lymph nodes were diagnosed as benign based on postoperative pathology examination. Furthermore, a total of 51 lymph nodes remained benign following repeated FNA and demonstrated no noteworthy alterations throughout the two-year ultrasound monitoring period. Notably, the metastatic group exhibited a higher prevalence of L/S ≤ 2 in lymph nodes compared to the benign group(p=0.008). The presence of calcification was observed to be significantly more frequent in lymph nodes of the metastatic group compared to that in the benign group (p = 0.000, subgroup analysis, calcified vs non-calcified, p = 0.000 < 0.017). Additionally, blood flow was found to be significantly richer in the metastatic group (p = 0.000, subgroup analysis, grade 0 VS 1, p = 0.007 < 0.017; grade 0 VS 2, 3, p = 0.000 < 0.017; grade 1 VS 2, 3, p = 0.006 <0.017).

Furthermore, postoperative lymph node dissection revealed that 77.8% of the metastatic group had HVLNM, whereas only 17.0% of the benign group had the same (p = 0.000). The median values of FNA-Tg in lymph nodes from the metastatic and benign groups were4376.5 (4791.50) IU/ml and 0.36 (2.38) IU/ml, respectively (P = 0.000). Among the 88 lymph nodes that also underwent FNA-TgAb testing, the median FNA-TgAb value was higher in the metastatic group compared to the benign group (12.15 (5.68) ng/mL and 26.10 (50.05) ng/mL, respectively, P = 0.000). Notably, lymph nodes displaying ultrasound characteristics indicative of metastasis possess a greater propensity for developing metastases compared to lymph nodes exhibiting structural abnormalities or reactive hyperplasia(p=0.000, comparison between subgroups, p < 0.017). Masses larger than 10mm in maximum diameter exhibit a higher likelihood of cervical LNM compared to those smaller than 10mm(p = 0.000, subgroup analysis, <10mm VS 10-20 mm, p = 0.002; <10mm VS ≥20mm, p = 0.000; 10-20 mm VS ≥20mm, p = 0.238 >0.017). The occurrence of thyroid peritoneal involvement is more probable in cases of lymph node metastasis.

There were no statistically significant differences observed between the two groups in terms of other serum parameters (such as sTg, sTSH, sTgAb), liquefaction in lymph nodes, slightly hyperechogenicity in the cortex, lymph node location partition, pathological types of thyroid cancer, and presence of HT (**Table 2**).

**Table 2:** Characteristic of lymph nodes.

Variables	Final Diagnosis		P
	Benign (n = 153)	Metastatic (n = 180)	
Location			
Zone1/2/3/4/5	5/6/51/83/3	1/5/58/102/1	0.146^b^
Zone 6/7	4/1	12/1	
TNM staging			0.000^b^
N0	53	0	
N1a	69	8	
N1b	31	172	
High volume lymph node metastasis			0.000 ^c^*
No	127	40	
Yes	26	140	
FNA			0.000 ^c^*
Benign	151	59	
Metastases	2	121	
FNA-Tg(cut-off value =16.1 ng/ml)			0.000 ^c^*
Benign	136	23	
Metastases	17	157	
Lateralization/suspicion (L/S)			0.008^c^*
≤2	61	98	
>2	92	82	
Liquefaction			0.059 ^b^
No	138	161	
Yes	8	17	
Suspicious	7	2	
Calcification			0.000 ^b^*
No	106	81	
Yes	33	77	
Suspicious	14	22	
Focal hyperechoic areas in cortex			0.053 ^c^*
No	147	164	
Yes	6	16	
Ultrasound diagnosis of lymph nodes			0.000 ^b^*
Benign or reactive hyperplasia	82	19	
Abnormal structure	32	22	
Metastases	39	139	
LN Alder Grade			0.000 ^b^*
0	95	77	
1	57	87	
2, 3	1	16	
Pathological types of thyroid cancer			0.430 ^b^
Classical PTC	134	167	
Classical + Follicular PTC variant	8	6	
Follicular PTC variant	8	5	
Follicular carcinoma	3	2	
sTg (ng/ml)^a^	13.2(21.39)	17.75(41.15)	0.052^d^
sTgAb (IU/ml) ^a^	15.08(51.05)	16.10(79.73)	0.971 ^d^
TSH(mU/L) ^a^	2.13(2.08)	2.18(1.97)	0.507 ^d^
FNA-Tg (ng/m) ^a^	0.375(2.45)	4376(4790.50)	0.000 ^d^*
FNA-TgAb (IU/ml) ^a^	12.15(5.68)	24.05(45.97)	0.000 ^d^*
Hashimoto’s thyroiditis	Benign (n = 134)^e^	Metastatic (n = 157)^e^	0.426 ^c^
No	96	119	
Yes	38	38	
Suspected malignant thyroid nodules	Benign (n = 128)^f^	Metastatic (n = 150)^f^	0.116^c^
single	80	80	
multiple	48	70	
Maximum diameter of the nodule			0.000^b^
<10mm	68	43	
10-20mm	44	67	
≥20mm	16	40	
Nodules invading the thyroid peritoneum			0,007^c^
NO	31	17	
Yes	97	133	

a Expressed as Mean (interquartile range).

b Fischer ‘s exact test.

c Chi-square test.

d Mann Whitney U test.

e 26 patients (42 lymph nodes) previously underwent total thyroidectomy.

f Out of the total cohort of 15 patients who had previously undergone partial thyroidectomy, 5(7 lymph nodes) sought medical attention due to suspicious thyroid nodules, while 10(13 lymph nodes) presented with suspicious lymph node metastases (as indicated by thyroid ultrasound, which did not detect any suspicious nodules in the remaining thyroid tissue).

*P Value of <0.05 was considered statistically significant.

### 3.3 The diagnostic efficacy of FNA, FNA-Tg, and their combined assays

**Figure 2:**
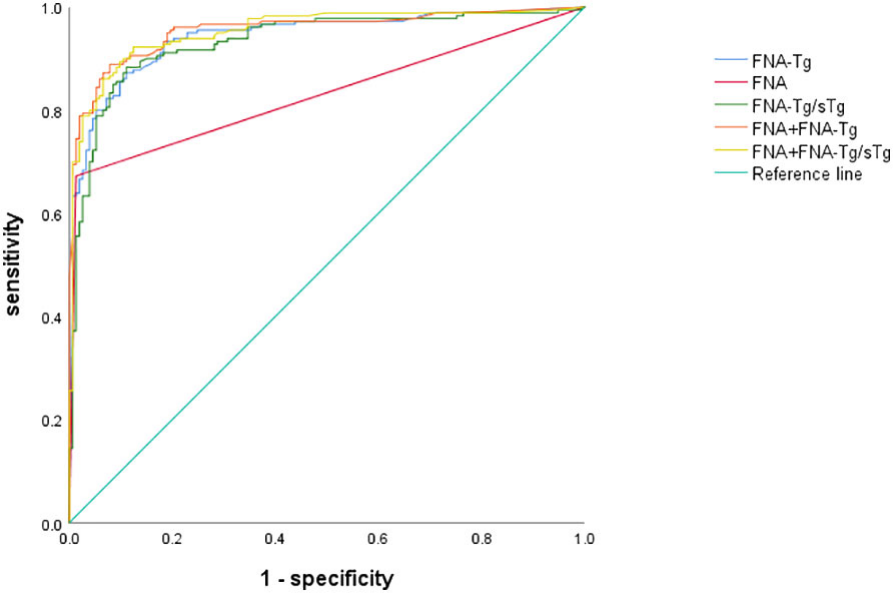
Comparison of area under the curve based on different diagnostic methods.

Among these, the FNA-Tg cut-off value of 16.1 ng/ml demonstrated the highest diagnostic accuracy(AUC= 0.942, 0.917-0.967), with a sensitivity of 87.2% and specificity of 89.9%. The PPV and NPV were calculated to be 90.2% and 85.5%, respectively. Furthermore, the cut-off value of FNA-Tg/sTg ratio was determined to be 1.41(AUC = 0.933, 0.905 – 0.961), exhibiting a sensitivity of 88.3% and specificity of 88.9%. The PPV and NPV for this ratio were calculated to be 90.3% and 86.1%, respectively. Additionally, a positive correlation was observed between sTg and FNA-Tg levels (Rs=0.318, P=0.000), while a negative correlation was found between sTgAb and FNA-Tg levels(Rs=-0.147, P=0.008) (**Table 3**). Diagnostic efficacy of each diagnostic tool and combined diagnostic method in **Table 4** and **Figure 2**.

**Table 3:** Relationship between serum parameters and FNA-Tg.

Variables	Rs	p
sTg	0.318	0.000*
TSH	0.017	0.761
sTgAb	-0.147	0.008*

*P Value of <0.05 was considered statistically significant.

**Table 4:** Diagnostic efficacy of each diagnostic tool and combined diagnostic method.

Diagnostic Tool	Diagnostic Value				
	Sensitivity (%)	Specificity (%)	PPV (%)	NPV (%)	AUC
FNA	67.2	98.7	98.4	71.9	0.830(0.917-0.967)
FNA-Tg	87.2	88.9	90.2	85.5	0.942(0.917-0.967)
FNA-Tg/sTg	88.3	88.9	90.3	86.1	0.933(0.905 – 0.961)
FNA+FNA-Tg	88.9	92.2	93.0	88.1	0.955 (0.933 – 0.977)
FNA+ FNA-Tg/sTg	92.2	87.6	90.2	90.5	0.954(0.932 – 0.976)

### 3.4 Analysis of possible influencing factors of FNA-Tg

In the context of lymph node metastatic staging, it was observed that lymph node FNA-Tg levels exhibited higher values in N1b compared to N0 and N1a(p = 0.000, subgroup analysis; N0 VS N1b, p = 0.000,N1a VS N1b, p = 0.000; N0 VS N1a, p = 0.999, Bonferroni correction). The optimal cut-off value for patients with N1b was higher than for those with N1a(1214.5 ng/ml, AUC=0.895(0.845-0.945) VS 115.5 ng/ml AUC=0.982(0.946-1.000), p=0.0065<0.05, DeLong test). In the presence of LNHVM, the levels of FNA-Tg were found to be elevated. When the L/S ratio of lymph nodes was ≤ 2, the levels of FNA-Tg were found to be higher compared to those with an L/S ratio>2. Patients diagnosed with HT exhibited higher levels of FNA-Tg compared to those without HT. The statistical analysis revealed a significant disparity in FNA-Tg levels between tumors with a maximum diameter below 10 mm and those exceeding 10 mm. However, no significant difference was observed between tumors measuring 10-20 mm and those surpassing 20 mm. Ultrasound examination revealed higher levels of FNA-Tg when lymph nodes exhibited liquefaction, calcification, and increased abundance. Furthermore, FNA-Tg levels were found to be higher in patients who were determined to have invaded the capsule based on postoperative pathology. Additionally, a positive correlation was observed between sTg and FNA-Tg levels. Conversely, FNA-Tg levels were lower in cases where sTgAb (+). Notably, no significant differences were observed in sTSH levels, the number of masses, pathological types of thyroid cancer, presence or absence of thyroid tissue, and presence or absence of slightly hyperechogenicity in the lymph node cortex (**Table 5**).

**Table 5:** Analysis of possible influencing factors of FNA-Tg.

Variables	Number	FNA-Tg^a^	p
TNM staging			0.000^e^
N0	53	0.24(1.74)	
N1a	77	0.46(3.41)	
N1b	203	1711(4969.80)	
High volume lymph node metastasis			0.000^d^*
Yes	167	972(4983.43)	
No	166	0.68(104.83)	
LN lateralization/suspicion (L/S)			0.000 ^d^*
>2	174	6.63(1686.49)	
≤2	159	409.00(4998.83)	
Liquefaction			0.007 ^d^*
No	299	19.2(4789.66)	
Yes	25	3556.00(4948.05)	
Suspicious	9	0.46(2500.85)	
Calcification			0.015 ^d^
No	187	5.70(4906.76)	
Yes	110	397.00(4997.33)	
Suspicious	36	16.45(549.37)	
Focal hyperechoic areas in cortex			0.2907 ^d^
No	311	25.1(4999.65)	
Yes	22	377.5(4747.23)	
LN Alder Grade			0.000 ^d^*
0	172	8.38(2007.05)	
1	144	202.50(4999.26)	
2, 3	17	5000(4983.50)	
TSH(mU/L) ^a^			0.084 ^d^
Low	38	253(4999.5)	
Normal	256	15.65(4287.21)	
High	39	99.6(4998.37)	
sTg(ng/ml)^a^			0.000 ^d^*
Low	76	4.325(569.96)	
Normal	211	30.2(4999.63)	
High	46	699(4989.84)	
sTgAb (IU/ml) ^a^			0.014 ^d^*
Normal	259	65.6(4999.56)	
High	74	5.635(399.80)	
Pathological types of thyroid cancer			0.468 ^d^
Classical PTC	301	30.2(4999.64)	
Classical + Follicular PTC variant	14	11.2(2788.93)	
Follicular PTC variant	13	49.8(2558.05)	
Follicular carcinoma	5	673(4988.2)	
Thyroid gland	N=291^b^		0.280 ^d^
Existence	249	25.1(4789.65)	
Absent	42	51.95(4999.4)	
Hashimoto’s thyroiditis	N=291^b^		0.016 ^d^*
No	215	99.60(4999.58)	
Yes	76	11.20(569.83)	
Number of Suspected malignant thyroid nodules	N=278^c^		0.428 ^d^
single	161	13.80(3957.73)	
multiple	118	45.00(4929.87)	
Maximum diameter of the nodule	N=278^c^		0.000 ^d^*
<10 mm	111	1.62(623.83)	
10≤, 20 mm	111	105.00(4999.62)	
≥20 mm	57	566.50(4990.62)	
Nodules invading the thyroid peritoneum	N=278^c^		0.004 ^d^*
No	48	1.94(181.106)	
Yes	231	105.00(4999.53)	

a Expressed as Mean (interquartile range).

b 26 patients (42 lymph nodes) previously underwent total thyroidectomy.

c Out of the total cohort of 15 patients who had previously undergone partial thyroidectomy, 5(7 lymph nodes) sought medical attention due to suspicious thyroid nodules, while 10(13 lymph nodes) presented with suspicious lymph node metastases (as indicated by thyroid ultrasound, which did not detect any suspicious nodules in the remaining thyroid tissue).

d Mann Whitney U test.

e Kruskal-Wallis test.

*P Value of <0.05 was considered statistically significant.

### 3.5 Diagnostic efficacy of FNA- TgAb

**Figure 3:**
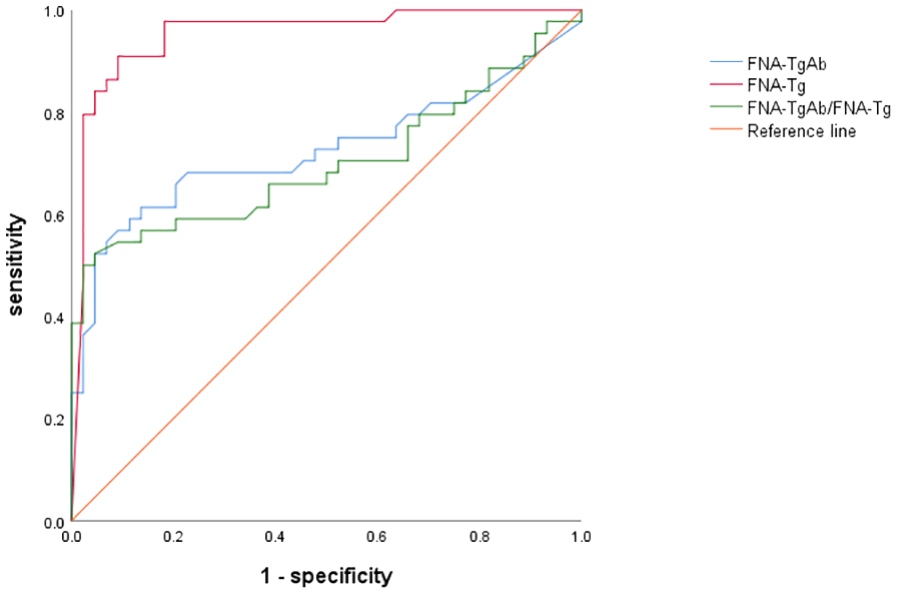
Comparison of area under the curve based on different diagnostic methods.

Furthermore, the AUC of FNA-TgAb and FNA-TgAb/s TgAb was smaller compared to that of FNA-Tg. However, it is worth noting that the specificity and positive predictive value of FNA-TgAb/s TgAb were high. The correlation between FNA-Tg and FNA-TgAb was found to be highly significant (P = 0.000; Spearman correlation coefficient = 0.537) (**Figure 3**, **Table 6**).

**Table 6:** Diagnostic efficacy of FNA-TgAb.

Diagnostic Tool	Diagnostic Value				
	Sensitivity (%)	Specificity (%)	PPV (%)	NPV (%)	AUC
FNA-TgAb	61.4	86.4	81.8	69.1	0.7268(0.613-0.838)
FNA-Tg	90.0	90.9	90.8	90.1	0.935(0.907-0.999)
FNA-TgAb/s TgAb	52.3	95.5	92.0	66.7	0.701(0.586-0.815)

## 4 Discussion

The concept of FNA-Tg was initially introduced by Pacini et al. in 1992, and it has been shown to be a valuable tool in the early detection of cervical LNM in patients with PTC (23). Since FNA-Tg is obtained during the FNA procedure, its implementation does not impose any additional discomfort on the patient, thereby reducing the reliance on individual expertise compared to cytology. However, the optimal cutoff value for FNA-Tg remains uncertain in current literature. Previous studies have proposed various cutoff values for FNA-Tg, ranging from 0.2 to 36 ng/mL (11).

This study demonstrates that a cutoff value of 16.1 ng/ml for FNA-Tg effectively diagnoses LNM. While FNA exhibits high specificity in diagnosing LNM, its sensitivity is limited. However, when combined with FNA-Tg, the sensitivity and NPV are improved. The AUC of FNA-Tg was greater than that of FNA(p <0.001, DeLong test).FNA-Tg exhibits high sensitivity and is effective in diagnosing both benign and malignant lymph nodes. The high specificity of FNA can be primarily attributed to its notable diagnostic efficacy in identifying malignant lymph nodes. However, it is important to note that in two cases, false positives occurred when FNA results initially indicated malignancy but were later pathologically confirmed as benign after surgical intervention. There was a significant positive correlation observed between FNA-Tg and sTg levels, with higher sTg levels corresponding to higher FNA-Tg levels. The cutoff value for FNA-Tg/sTg was determined to be 1.41.In comparison to other diagnostic tests, the combination of FNA and FNA-Tg/sTg demonstrated the highest sensitivity, which aligns with the findings of Liu et al. (11).

This study may be the first to report the correlation between HVLNM and lymph node FNA-Tg levels. There are studies (24–26) demonstrating that several factors, including younger age, male gender, tumor size, extrathyroidal extension, multifocality, presence of microcalcification, peritumoral infiltration, and abundant blood flow, were found to be independent clinicopathological risk factors associated with HVLNM.According to a study conducted by Hui Huang (27) et al., it was found that patients without concomitant HT exhibited a significantly higher risk of HVLNM compared to those with concomitant HT. Furthermore, the presence of HVLNM is associated with higher FNA-Tg levels, suggesting that patients with elevated FNA-Tg levels may necessitate comprehensive lymph node dissection to ensure the removal of hidden metastatic lymph nodes in clinical settings.

There was no statistical difference in the distribution of locations between benign and malignant lymph nodes. Interestingly, the optimal cut-off value for patients with N1b was higher than for those with N1a. One potential explanation for this phenomenon is that thyroid cancer, upon developing extra-thyroidal infiltration, exhibits a tendency to initially metastasize to the central zone lymph nodes, followed by subsequent metastasis to the cervical lateral zone lymph nodes. Consequently, patients diagnosed with N1b demonstrate a greater extent of tumour cell infiltration when metastasis occurs in the cervical lateral zone lymph nodes, resulting in elevated levels of FNA-Tg cut-off values.

Currently, there is ongoing debate regarding the impact of sTSH, sTg, sTgAb, and the presence of thyroid gland *in situ* on FNA-Tg levels. Our study demonstrates a positive correlation between sTg and FNA-Tg levels, aligning with the findings of Konca et al (13). Therefore, elevated levels of sTg are associated with higher FNA-Tg cutoff values (8, 28). The levels of FNA-Tg were observed to be lower in individuals with sTgAb (+). Jia et al. (22) also discovered a negative correlation between serum TgAb levels and FNA-Tg levels in the central LN (Rs =-0.232,p = 0.028). These findings align with previous research (15, 29), suggesting that elevated sTgAb levels may impede accurate FNA-Tg measurements.

Limited research has been conducted on the correlation between HT and FNA-Tg levels in lymph nodes. The findings of this study indicate that suspicious lymph nodes with HT exhibit lower FNA-Tg levels compared to those without HT. The present research have proposed a correlation between HT and an augmented susceptibility to thyroid cancer, coupled with a reduced occurrence of LNM, aligning with the outcomes of our study (30). Our investigation revealed that FNA-Tg levels were reduced in the presence of positive serum TgAb. This observation may be attributed to elevated sTgAb levels in HT patients.

Ultrasound examination revealed higher FNA-Tg levels in cases where lymph nodes exhibited L/S ≤ 2, liquefaction, calcification, and increased blood flow. This observation suggests a positive association between the malignancy of lymph nodes and their FNA-Tg levels. Degertekin et al’s study additionally demonstrated that FNA-Tg levels rise as lymph nodes exhibit a rounded shape on ultrasonography (13). Further investigations are warranted to explore the relationship between ultrasonographic characteristics of lymph nodes and FNA-Tg levels.

Factors such as TSH level, number of masses, pathological type, presence or absence of thyroid tissue, location of lymph node distribution, and presence or absence of slightly hyper-echogenicity in lymph nodes did not exert a statistically significant influence on FNA-Tg. Our study aligns with the findings of Wang, Jiahui et al. (31), who determined that the status of thyroid tissue does not impact the accuracy of FNA-Tg in diagnosing LNM. The two AUC (0.969 and 0.987) showed no significant difference. However, other studies (32) discovered that patients with thyroid tissue (prior to primary surgery or after lobectomy) exhibited higher FNA-Tg cutoff values compared to patients without thyroid tissue (55.99 vs. 9.7 ng/mL, respectively). While the influence of thyroid presence or absence requires further clarification, it is crucial to consider the integrity of thyroid tissue in clinical practice.

Furthermore, our investigation revealed that suspicious lymph nodes exhibited heightened levels of FNA-Tg when the maximum tumor diameter exceeded 10 mm, as opposed to cases of minimal thyroid cancer. Consequently, a more cautious approach is warranted when evaluating lymph nodes in the context of minimal thyroid cancer within clinical.

Our study has demonstrated that FNA-TgAb exhibits limited diagnostic efficacy in detecting LNM in DTC. However, it is worth noting that both the specificity and PPV of FNA-TgAb and FNA-TgAb/s TgAb were found to be high. Additionally, we observed a positive correlation between FNA-Tg and FNA-TgAb (P = 0.000; Spearman correlation coefficient = 0.537), which aligns with the findings reported by Liu, Qianhui et al (33). Nevertheless, further follow-up studies are necessary to fully understand the impact of FNA-TgAb on the diagnostic efficacy of FNA-Tg in detecting LNM in DTC.

This study is subject to certain limitations. Firstly, it should be noted that this study adopts a retrospective analysis approach, which introduces a potential selection bias. Secondly, due to the retrospective nature of the study, it was not possible to guarantee the exact correspondence between the lymph nodes that were biopsied and subsequently surgically removed. The cases, where the biopsied lymph nodes were situated at the same neck level, and exhibited similar size, and were successfully removed, were deemed to be the same (34). The concordance of lymph nodes was determined through the utilization of hook needle localization, staining, or postoperative aspiration of FNA-Tg. Following this, we will assess the agreement of lymph nodes through the utilization of hook needle localization, staining, or postoperative aspiration of FNA-Tg. Furthermore, it is imperative to expand the sample size by including more patients and incorporating a wider range of cases beyond classical PTC, while also enhancing the precision of the findings.

## 5 Conclusion

In summary, this investigation proposes that the combination of FNA and FNA-Tg holds significant diagnostic value in the identification of LNM in DTC.Factors such as TNM stage indicated N1b, the presence of HVLNM, lymph node L/S, liquefaction, calcification, tumor diameter, the presence of HT, sTg, and sTgAb can influence FNA-Tg levels.

## Data availability statement

The raw data supporting the conclusions of this article will be made available by the authors, without undue reservation.

## Ethics statement

The studies involving humans were approved by Ethics Committee of West China Hospital, Sichuan University. The studies were conducted in accordance with the local legislation and institutional requirements. Written informed consent for participation was not required from the participants or the participants’ legal guardians/next of kin in accordance with the national legislation and institutional requirements.

## Author contributions

XS: Writing – original draft, Formal analysis, Data curation, Conceptualization. LS: Writing – original draft, Data curation, Conceptualization. CY: Writing – original draft, Data curation, Conceptualization. BM: Writing – review & editing.

## References

[1] Mao Y Xing M. Recent incidences and differential trends of thyroid cancer in the USA. Endocrine-Related Cancer. (2016) 23:313–22. doi: 10.1530/ERC-15-0445 PMC4891202 26917552

[2] Sherma SI. Thyroid carcinoma. Lancet. (2003) 361:501–11. doi: 10.1016/S0140-6736(03)12488-9 12583960

[3] Grani G Fumarola A. Thyroglobulin in lymph node fine-needle aspiration washout: a systematic review and meta-analysis of diagnostic accuracy. J Clin Endocrinol Metab. (2014) 99:1970–82. doi: 10.1210/jc.2014-1098 24617715

[4] Hay ID Hutchinson ME Gonzalez-Losada T McIver B Reinalda ME Grant CS. Papillary thyroid microcarcinoma: A study of 900 cases observed in a 60-year period. Surgery. (2008) 144:980–8. doi: 10.1016/j.surg.2008.08.035 19041007

[5] Zhao H Li H. Meta-analysis of ultrasound for cervical lymph nodes in papillary thyroid cancer: Diagnosis of central and lateral compartment nodal metastases. Eur J Radiol. (2019) 112:14–21. doi: 10.1016/j.ejrad.2019.01.006 30777203

[6] Jiang HJ Hsiao PJ. Clinical application of the ultrasound-guided fine needle aspiration for thyroglobulin measurement to diagnose lymph node metastasis from differentiated thyroid carcinoma-literature review. Kaohsiung J Med Sci. (2020) 36:236–43. doi: 10.1002/kjm2.12173 PMC11896569 31909556

[7] Orija IB Hamrahian AH Reddy SSK. Management of nondiagnostic thyroid fine-needle aspiration biopsy: survey of endocrinologists. Endocrine Pract. (2004) 10:317–23. doi: 10.4158/ep.10.4.317 15760774

[8] Moon JH Kim YI Lim JA Choi HS Cho SW Kim KW. Thyroglobulin in washout fluid from lymph node fine-needle aspiration biopsy in papillary thyroid cancer: large-scale validation of the cutoff value to determine Malignancy and evaluation of discrepant results. J Clin Endocrinol Metab. (2013) 98:1061–8. doi: 10.1210/jc.2012-3291 23393171

[9] Zhu XH Zhou JN Qian YY Yang K Wen QL Zhang QH. Diagnostic values of thyroglobulin in lymph node fine-needle aspiration washout: a systematic review and meta-analysis diagnostic values of FNA-Tg. Endocr J. (2020) 67:113–23. doi: 10.1507/endocrj.EJ18-0558 31723088

[10] Haugen BR Alexander EK Bible KC Doherty GM Mandel SJ Nikiforov YE. 2015 American thyroid association management guidelines for adult patients with thyroid nodules and differentiated thyroid cancer: the american thyroid association guidelines task force on thyroid nodules and differentiated thyroid cancer. Thyroid. (2016) 26:1–133. doi: 10.1089/thy.2015.0020 26462967 PMC4739132

[11] Liu R-B Zhou D-L Xu B-H Yang X-H Liu Q Zhang X. Comparison of the diagnostic performances of US-guided fine needle aspiration cytology and thyroglobulin measurement for lymph node metastases in patients with differentiated thyroid carcinoma: a meta-analysis. Eur Radiol. (2020) 31:2903–14. doi: 10.1007/s00330-020-07400-9 33125564

[12] Boi F Baghino G Atzeni F Lai ML Faa G Mariotti S. The diagnostic value for differentiated thyroid carcinoma metastases of thyroglobulin (Tg) measurement in washout fluid from fine-needle aspiration biopsy of neck lymph nodes is maintained in the presence of circulating anti-tg antibodies. J Clin Endocrinol Metab. (2006) 91:1364–9. doi: 10.1210/jc.2005-1705 16434461

[13] Konca Degertekin C Yalcin MM Cerit T Ozkan C Kalan I Iyidir OT. Lymph node fine-needle aspiration washout thyroglobulin in papillary thyroid cancer: Diagnostic value and the effect of thyroglobulin antibodies. Endocr Res. (2016) 41:281–9. doi: 10.3109/07435800.2016.1141936 26905960

[14] Jeon SJ Kim E Park JS Son KR Baek JH Kim YS. Diagnostic benefit of thyroglobulin measurement in fine-needle aspiration for diagnosing metastatic cervical lymph nodes from papillary thyroid cancer: correlations with US features. Korean J Radiol. (2009) 10:106–11. doi: 10.3348/kjr.2009.10.2.106 PMC2651448 19270855

[15] Jo K Kim MH Lim Y Jung SL Bae JS Jung CK. Lowered cutoff of lymph node fine-needle aspiration thyroglobulin in thyroid cancer patients with serum anti-thyroglobulin antibody. Eur J Endocrinol. (2015) 173:489–97. doi: 10.1530/EJE-15-0344 26208979

[16] Tang S Buck A Jones C Sara Jiang X. The utility of thyroglobulin washout studies in predicting cervical lymph node metastases: One academic medical center's experience. Diagn Cytopathol. (2016) 44:964–8. doi: 10.1002/dc.23554 27546053

[17] Li QK Nugent SL Straseski J Cooper D Riedel S Askin FB. Thyroglobulin measurements in fine-needle aspiration cytology of lymph nodes for the detection of metastatic papillary thyroid carcinoma. Cancer Cytopathol. (2013) 121:440–8. doi: 10.1002/cncy.21285 23495036

[18] Randolph GW Duh Q-Y Heller KS LiVolsi VA Mandel SJ Steward DL. The prognostic significance of nodal metastases from papillary thyroid carcinoma can be stratified based on the size and number of metastatic lymph nodes, as well as the presence of extranodal extension. Thyroid. (2012) 22:1144–52. doi: 10.1089/thy.2012.0043 23083442

[19] Adam MA Pura J Goffredo P Dinan MA Reed SD Scheri RP. Presence and number of lymph node metastases are associated with compromised survival for patients younger than age 45 years with papillary thyroid cancer. J Clin Oncol. (2015) 33:2370–5. doi: 10.1200/JCO.2014.59.8391 26077238

[20] Adler DD Carson PL Rubin JM Quinn-Reid D. Doppler ultrasound color flow imaging in the study of breast cancer: preliminary findings. Ultrasound Med Biol. (1990) 16:553–9. doi: 10.1016/0301-5629(90)90020-D 2238263

[21] Kim K Bae JS Kim JS. Measurement of thyroglobulin level in lateral neck lymph node fine needle aspiration washout fluid in papillary thyroid cancer. Gland Surg. (2021) 10:2686–94. doi: 10.21037/gs PMC8514304 34733718

[22] Jia X Wang Y Liu Y Wang X Yao X Tao R. Thyroglobulin measurement through fine-needle aspiration for optimizing neck node dissection in papillary thyroid cancer. Ann Surg Oncol. (2022) 29:88–96. doi: 10.1245/s10434-021-10549-2 34386915 PMC8677638

[23] Pacini F Fugazzola L Lippi F Ceccarelli C Centoni R Miccoli P. Detection of thyroglobulin in fine needle aspirates of nonthyroidal neck masses: a clue to the diagnosis of metastatic differentiated thyroid cancer. J Clin Endocrinol Metab. (1992) 74:1401–4. doi: 10.1210/jcem.74.6.1592886 1592886

[24] Wang Z Gui Z Wang Z Huang J He L Dong W. Clinical and ultrasonic risk factors for high-volume central lymph node metastasis in cN0 papillary thyroid microcarcinoma: A retrospective study and meta-analysis. Clin Endocrinol. (2022) 98:609–21. doi: 10.1111/cen.14834 36263602

[25] Zhang L Wang P Li K Xue S. A novel nomogram for identifying high-risk patients among active surveillance candidates with papillary thyroid microcarcinoma. Front Endocrinol. (2023) 14. doi: 10.3389/fendo.2023.1185327 PMC10541211 37780614

[26] Xue S Zhang L Pang R Wang P Jin M Guo L. Predictive factors of central-compartment lymph node metastasis for clinical N0 papillary thyroid carcinoma with strap muscle invasion. Front Endocrinol. (2020) 11. doi: 10.3389/fendo.2020.00511 PMC7506113 33013682

[27] Huang H Liu Y Ni S Liu S. A prediction model for identifying high-risk lymph node metastasis in clinical low-risk papillary thyroid microcarcinoma. BMC Endocrine Disord. (2023) 23:260–71. doi: 10.1186/s12902-023-01521-0 PMC10680325 38012653

[28] Lee JH Lee HC Yi HW Kim BK Bae SY Lee SK. Influence of thyroid gland status on the thyroglobulin cutoff level in washout fluid from cervical lymph nodes of patients with recurrent/metastatic papillary thyroid cancer. Head Neck. (2016) 38:E1705–12. doi: 10.1002/hed.24305 26614648

[29] Jeon MJ Park JW Han JM Yim JH Song DE Gong G. Serum antithyroglobulin antibodies interfere with thyroglobulin detection in fine-needle aspirates of metastatic neck nodes in papillary thyroid carcinoma. J Clin Endocrinol Metab. (2013) 98:153–60. doi: 10.1210/jc.2012-2369 23144473

[30] Jara SM Carson KA Pai SI Agrawal N Richmon JD Prescott JD. The relationship between chronic lymphocytic thyroiditis and central neck lymph node metastasis in North American patients with papillary thyroid carcinoma. Surgery. (2013) 154:1272–82. doi: 10.1016/j.surg.2013.07.021 24238047

[31] Wang J Jiang X Xiao G Zhou W Hu Y. Excellent diagnostic performance of FNA-Tg in detecting lymph nodes metastases from papillary thyroid cancer. Future Oncol. (2020) 16:2735–46. doi: 10.2217/fon-2020-0213 32812450

[32] Zhao H Wang Y Wang M-j Zhang Z-h Wang H-r Zhang B. Influence of presence/absence of thyroid gland on the cutoff value for thyroglobulin in lymph-node aspiration to detect metastatic papillary thyroid carcinoma. BMC Cancer. (2017) 17:296–303. doi: 10.1186/s12885-017-3296-3 28454525 PMC5410021

[33] Liu Q Mao L Zhang Z Li G Song H. Diagnostic efficacy of FNA-tg in DTC cervical LN metastasis and its impact factors: A large retrospective study. J Clin Endocrinol Metab. (2023) 108(12):3311–3319. doi: 10.1210/clinem/dgad335 37279938

[34] Xiao J Meng S Zhang M Li Y Yan L Li X. Optimal method for detecting cervical lymph node metastasis from papillary thyroid cancer. Endocrine. (2022) 79:342–8. doi: 10.1007/s12020-022-03213-6 36472754

